# Assessment of Serum Dynamic Thiol/Disulfide Homeostasis and Oxidative/Nitrosative Stress in Patients with Crohn’s Disease

**DOI:** 10.5152/tjg.2024.23519

**Published:** 2024-11-25

**Authors:** Ancel Aysun Bağdaş, Sezgin Barutçu, Ahmet Saracaloğlu, Abdullah Tuncay Demiryürek

**Affiliations:** 1 Department of Gastroenterology, aziantep University Faculty of Medicine, Gaziantep, Türkiye; 2 Department of Medical Pharmacology, Gaziantep University Faculty of Medicine, Gaziantep, Türkiye

**Keywords:** Crohn’s disease, nitrosative stress, oxidative stress

## Abstract

**Background/Aims:**

Crohn’s disease (CD) is a major subtype of chronic relapsing inflammatory gastrointestinal disorders. In this study, we assessed the possible contributions of serum oxidative/nitrosative stress and dynamic thiol/disulfide homeostasis to CD pathogenesis.

**Materials and Methods:**

Patients with active CD (A-CD) at onset (n = 38), CD patients in the remission (R-CD) (n = 38), and healthy controls (n = 38) were prospectively included in this study. Serum oxidative/nitrosative parameters as well as total thiol and native thiol levels were analyzed.

**Results:**

We observed significant augmentation in nitric oxide (NO) levels in both A-CD and R-CD patients compared to healthy controls. We detected marked reductions in the 3-nitrotyrosine levels in the patient groups. Glutathione, glutathione peroxidase, and myeloperoxidase levels were observed to be significantly lower in both the active and remission groups (*P* < .001). In the A-CD group, native thiol (*P* < .001) and total thiol (*P* < .01) levels were lower, and disulfide levels were higher than those of the control group (*P* < .01), while the native thiol/total thiol ratio was reduced and disulfide/total thiol (*P* < .001) and disulfide/native thiol (*P* < .001) ratios were elevated. Remarkably, no change in dynamic thiol/disulfide homeostasis was found in the R-CD group.

**Conclusion:**

Our results showed increased serum NO levels and decreased antioxidant enzymes, particularly during the active phase of CD. Determination of thiol/disulfide homeostasis could help differentiate between the active and remission phases of the disease. Thiol/disulfide parameters can be used as biomarkers for A-CD.

**Main Points**

- The finding of low native thiol and high disulfide levels in patients with active Crohn’s disease (CD) suggests the involvement of dynamic thiol/disulfide homeostasis in CD.
- Marked augmentation in nitric oxide (NO) levels indicates the involvement of nitrosative stress in CD.
- Significant reductions in serum glutathione and glutathione peroxidase levels indicate that a depressed antioxidant defense contributes to the pathogenesis of CD.
- Differentiation between the active and remission phases of CD appears to be possible through the analysis of thiol/disulfide homeostasis.

## Introduction

Crohn’s disease (CD) is a complex remitting–relapsing inflammatory bowel disease (IBD) with a multifactorial origin causing uncontrolled and inappropriate immune response with impaired gut mucosal barrier homeostasis.^1^ The etiopathogenesis of CD involves immunological, genetic, environmental, and microbiological factors that interact to promote immune imbalance and alter intestinal homeostasis, causing the emergence of chronic inflammation. Epigenetic factors also play crucial roles in the pathogenesis of CD.^2^ The main clinical manifestations of CD include diarrhea, abdominal pain, fatigue, fever, nausea, vomiting, and anorexia. Extraintestinal manifestations with mucocutaneous involvement are also evident in CD.^3^ With a rapidly rising trend worldwide, the annual incidence of CD has been estimated at 0.06–29.3 cases per 100,000 people.^4^

Unfortunately, no radical cure for CD is currently available. Due to the autoimmune nature of CD, most of the therapies utilized for this disorder aim to diminish the dysregulated inflammatory response. Surgical intervention for the treatment of Crohn’s-related complications is generally reserved for patients who are in an acute, life-endangering state or who have not responded to pharmacologic therapies. Moreover, surgery is not curative, and patients require ongoing therapy after surgery to prevent disease recurrence.^1,4^

Although the etiological factors for CD are not completely understood, evidence suggests that oxidative stress may be a principal effector mechanism causing tissue injury and cellular damage in the initiation and progression of CD.^5^ The combination of augmented reactive oxygen species (ROS)/reactive nitrogen species (RNS) formation and reduced antioxidant status describes several pathological aspects of CD.^5^ Furthermore, inflammation increases oxidative stress by inducing the ROS/RNS-producing systems, along with myeloperoxidase (MPO) enzyme release from inflammatory cells.^5^ Both intestinal epithelial and immune cells generate proinflammatory cytokines that stimulate the formation of ROS/RNS during mucosal inflammation. These reactive species are associated with the progression and/or initiation of CD.^1,5^

Since thiols (sulfydryl groups) are primary substrates for ROS and are rapidly oxidized by ROS metabolites, plasma-free thiols are principally viewed as a strong measure of the *in vivo *redox state. Quantifying systemic redox balance in CD could be an appropriate and minimally invasive strategy for monitoring disease activity.^6^ However, our understanding of the involvement of thiol/disulfide homeostasis in the pathophysiology of CD is limited. Therefore, the objective of this study was to identify the serum oxidative and nitrosative stress states and assess the role of dynamic thiol/disulfide homeostasis in CD.

## Materials and Methods

### Study population

Using consecutive sampling, a total of 76 patients with active CD at onset (A-CD) (n = 38, age median (range) = 31 (19-68) years old) or with CD in remission (R-CD) (n = 38, age median (range) = 35.5 (21-74) years old) who were admitted to the Gastroenterology Department of the Gaziantep University Hospital between November 2021 and October 2022 were prospectively recruited to participate in this research. This study was approved by Gaziantep University Clinical Research Ethics Committee (approval number: 2021/302, date: November 17, 2021). Written informed consent was obtained from the patients who participated in this study. Prior to beginning drug treatment, serum measurements were taken from patients in the A-CD group. The R-CD group comprised patients who had achieved clinical remission after the flare-up phase of the disease. The control group was composed of 38 age-matched healthy volunteers (n = 38, age median (range) = 31 (25-55) years old), selected from the hospital’s staff and their families. CD patients with any evidence of renal failure, liver disease, bowel obstruction, active gastrointestinal bleeding, abdominal abscess, diabetes, coronary or peripheral artery disease, hypertension, or pregnancy were excluded from the study. In response to ethical concerns, patients in the R-CD group were kept on their current drug therapies. Participants in the control group were excluded if there was documentation of bacterial or viral infection; a family history of CD; diagnosed genetic, neurologic, liver, or psychiatric disease; anti-inflammatory drug usage; or the presence of inflammatory disorders.

Among the patients in the R-CD group, the CD types were 58% inflammatory, 16% obstructive, and 26% fistulizing, and in the A-CD group, 71% were inflammatory, 16% obstructive, and 13% fistulizing. In terms of locations of disease involvement, 37% had ileocolonic, 51% ileal, and 12% colonic involvement in the R-CD group, and 48% had ileocolonic, 37% ileal, and 15% colonic involvement in the A-CD group.

CD diagnosis was based upon colonoscopic/endoscopic data, histopathological evaluation, laboratory findings, and clinical signs.^7^ While endoscopic activity scores were not universally obtainable due to patient preference, the Harvey-Bradshaw Index (HBI) was used to assess clinical disease activity.^8^ The HBI is subclassified as those in remission with a calculated score of <5 or active disease (mild 5-7, moderate 8-16, severe >16). This study received approval from the clinical ethics committee of Gaziantep University (reference number: 2021/302). All participants provided informed consent before enrollment, and procedures adhered to the principles of the Declaration of Helsinki.

### Blood samples

Blood samples were collected via venipuncture after an overnight fast. Samples were placed in serum separator tubes, allowed to clot, and centrifuged for 10 min at 1500 *g*. The resulting serum was immediately stored at −80°C until analysis. All parameters were analyzed in a single-day experiment series to ensure consistent and uniform assay conditions. Samples were thawed and assayed immediately to prevent potential declines in enzyme activity. Additionally, standard laboratory analyses were performed for all patients.

### Thiol/Disulfide detection

Serum native thiol (-SH) and total thiol (-SH + -S-S-) levels were measured using a commercially available kits (Rel Assay Diagnostics, Mega Tip Ltd, Gaziantep, Türkiye) according to established protocols.^9^ Briefly, specimens were incubated with 1.9 mM 5,5’-dithio-bis-(2-nitrobenzoic acid) (DTNB, Ellman’s Reagent) in phosphate buffer for 20 minutes at room temperature. Free thiol groups were then quantified by absorbance readings using a microplate reader (Epoch Microplate Spectrophotometer, BioTek Instruments, Winooski, VT, USA). Dynamic disulfide (-S-S) levels were calculated as half the difference between total and native thiol values.

### Nitric Oxide (NO) Level Detection

Serum NO levels were determined using a previously described chemiluminescence method.^9^ Briefly, the serum samples were treated with absolute ethanol, and incubated for 30 minutes at 0^°^C followed by centrifugation at 20 800 *g* for 5 minutes. An NO analyzer (Model 280i NOA, Sievers Instruments, Boulder, CO, USA) was utilized to assess NO levels in the supernatants using vanadium trichloride as the reducing agent. NO levels were calculated from the standard curve produced by sodium nitrate. The NOAnalysis^TM^ software (version 3.21, Sievers, Boulder, CO, USA) was used for data recording and analysis.

### ELISA Measurements

Serum 3-nitrotyrosine (3-NT) levels were measured using a commercially available ELISA kit (Cat. No. CK-bio-10045, Coon Koon Biotech Co. Ltd., Shanghai, China). The concentrations of glutathione (GSH) (Cat. No. 201-12-1463), glutathione peroxidase (GSH-Px) (Cat. No. 201-12-0726), and MPO (Cat. No. 201-12-0881) in the serum were detected using commercially available human ELISA kits (Sunredbio Technology Co. Ltd., Shanghai, China). Absorbance readings for all assays were obtained at 450 nm using a microplate reader.

### Statistical Analysis

Data were given as mean ± standard error of mean (SEM), standard deviation (SD), or percentages. Normality was assessed using the Kolmogorov-Smirnov test. The Mann–Whitney U test was utilized for data with non-normal distribution. Otherwise, an unpaired Student’s t-test was applied to compare 2 groups of normally distributed data. The ANOVA test was utilized to compare more than 2 independent groups, followed by a *post hoc* Student-Newman-Keuls test for multiple comparisons. When assumptions of normality were not fulfilled, the Kruskal-Wallis test followed by Dunn’s *post hoc* test was applied for comparing the means of more than 2 groups. The chi-square or Fisher’s exact tests were utilized to analyze the categorical data. Depending on the distribution of data, correlation analyses were performed with Spearman’s rank or Pearson correlation tests. GraphPad Instat version 3.05 (GraphPad Software Inc., San Diego, CA, USA) or IBM SPSS version 22.0 (SPSS Inc., Chicago, IL, USA) software was used, and *P* values < 0.05 were accepted as significant. Power analysis conducted using G*Power 3.1.9.4 (Heinrich Heine University, Dusseldorf, Germany) indicated a minimum sample size of 37 subjects per group.

## Results

Table 1 summarizes the clinical, laboratory, and demographic data for the CD patient groups and controls. Compared to the control group, gender, average age, smoking status, white blood cell counts, mean corpuscular volume, hemoglobin, glucose, creatinine, urea, aspartate aminotransferase, gamma-glutamyl transferase, alanine aminotransferase, ferritin, folic acid, vitamin B12, and serum globulin levels were similar (*P* > .05 for all). Platelet count was markedly elevated in the A-CD group compared to the control and R-CD groups. C-reactive protein (CRP) values, alkaline phosphatase activity, and erythrocyte sedimentation rate were significantly higher in both A-CD and R-CD groups compared to controls. However, serum iron, total iron-binding capacity, and albumin levels were markedly lower in both A-CD and R-CD groups compared to controls (Table 1).

**Figure 1.**
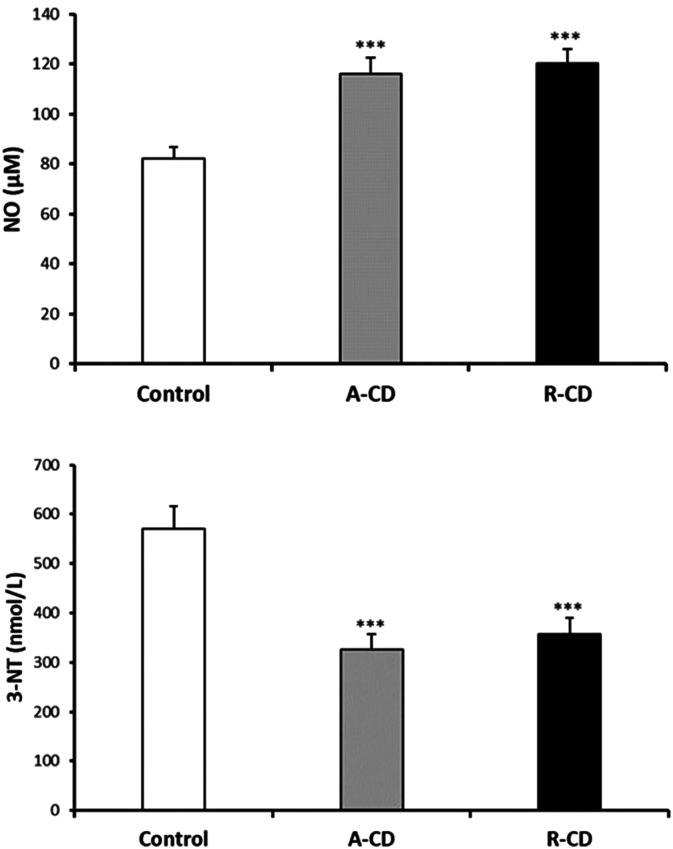
Serum nitric oxide (NO) and 3-nitrotyrosine (3-NT) levels between controls, active Crohn’s disease (A-CD) at onset, and CD patients in the remission (R-CD) groups (n = 38 for each group). Values are given as mean ± SEM, ***P < .001 compared to control group.

**Figure 2.**
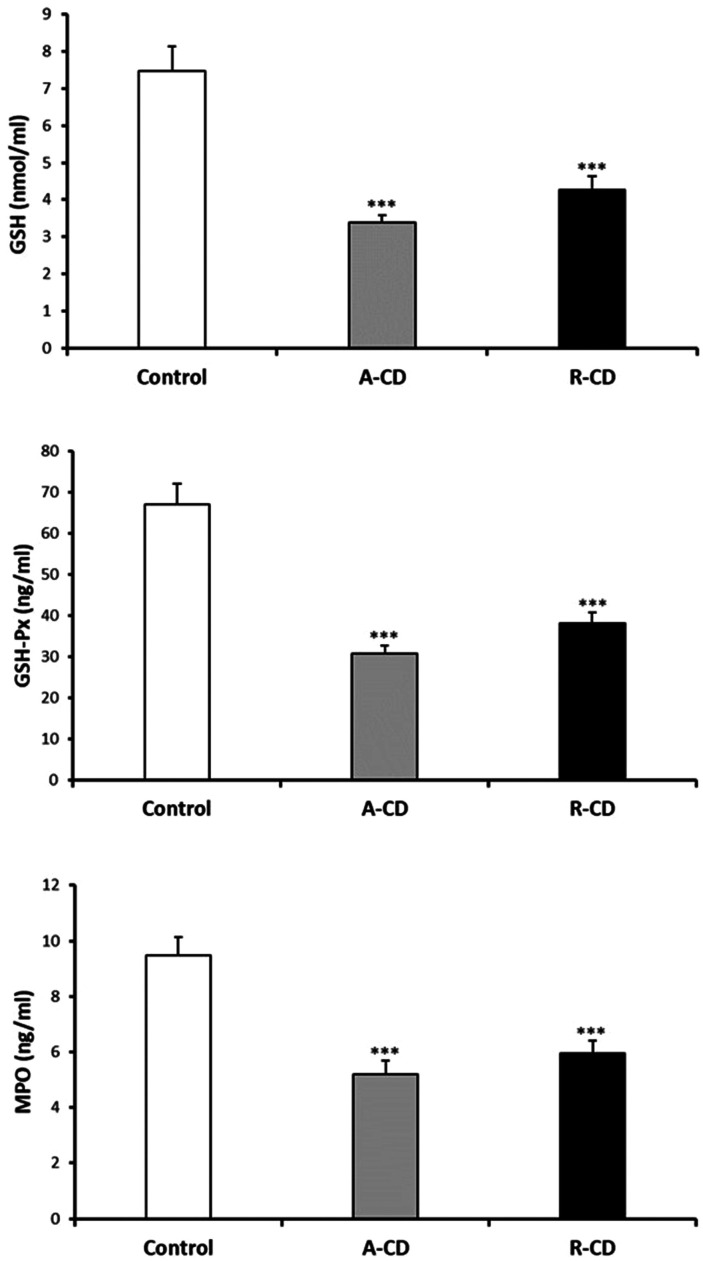
Serum glutathione (GSH), glutathione peroxidase (GSH-Px), and myeloperoxidase (MPO) levels between controls, active Crohn’s disease (A-CD) at onset, and CD patients in the remission (R-CD) groups (n = 38 for each group). Values are given as mean ± SEM, ***P < .001 compared to control group.

**Figure 3.**
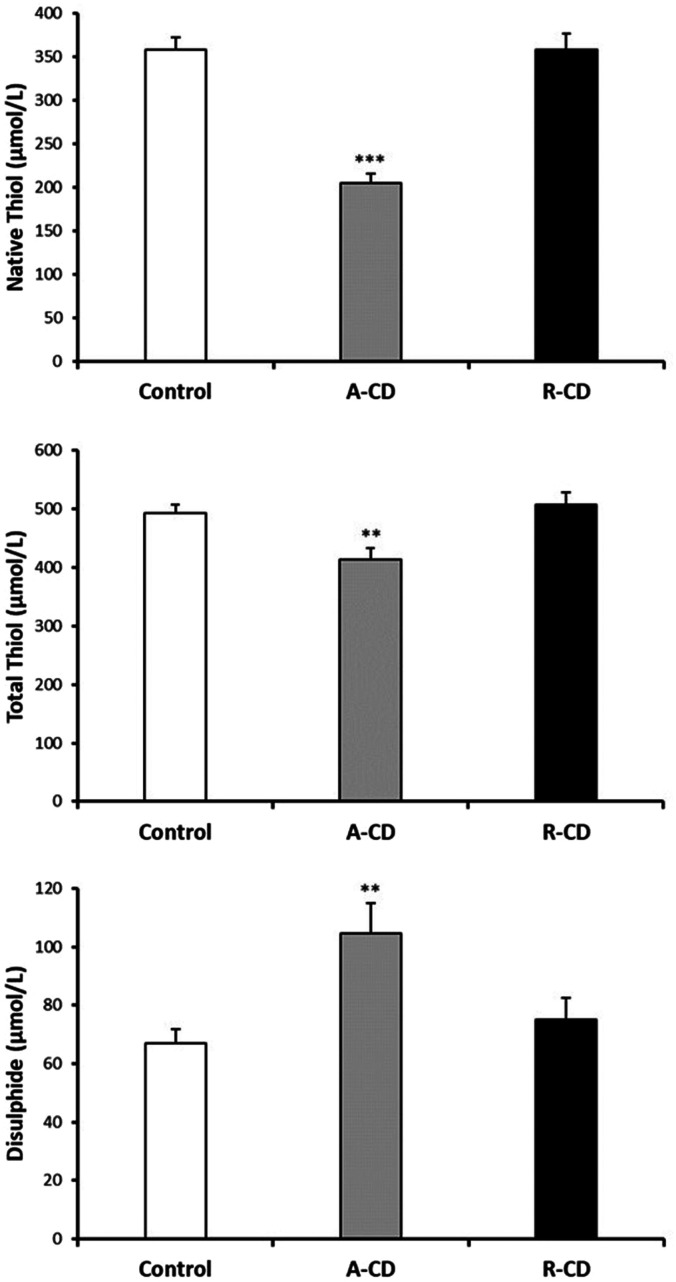
Serum native thiol, total thiol, and disulfide levels between controls, active Crohn’s disease (A-CD) at onset, and CD patients in the remission (R-CD) groups (n = 38 for each group). Values are given as mean ± SEM, **P < .01 and ***P < .001 compared to control group.

**Figure 4.**
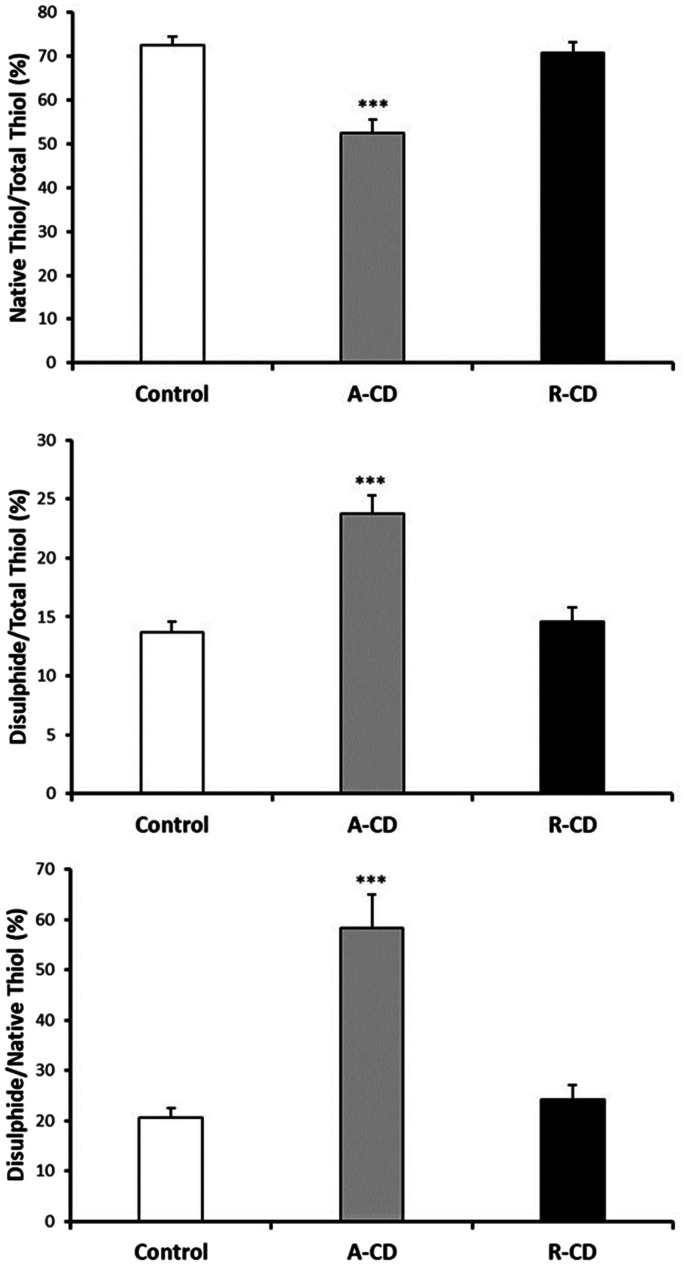
Native thiol/total thiol, disulfide/total thiol, and disulfide/native thiol ratios between controls, active Crohn’s disease (A-CD) at onset, and CD patients in the remission (R-CD) groups (n = 38 for each group). Values are given as mean ± SEM, ***P < .001 compared to control group.

3-NT and NO levels were evaluated as the nitrosative stress markers. Serum NO levels were significantly elevated in both A-CD and R-CD patients compared to healthy controls (Figure 1). Serum NO levels were significantly elevated in both A-CD and R-CD patients compared to healthy controls. There were no changes between A-CD and R-CD patients in terms of NO and 3-NT levels (*P* > .05, Figure 1). Diminished serum MPO, GSH, and GSH-Px were recorded in CD patients (Figure 2). Although there were significant attenuations in serum native thiol (*P* < .001) and total thiol (*P* < .01) levels, a marked elevation in disulfide levels (*P* < .01) was found in the A-CD group compared to controls (Figure 3). These values were not changed significantly in the R-CD group and showed no differences compared to controls. Our data demonstrated that the native thiol/total thiol ratio was declined (*P* < .001), but disulfide/total thiol (*P* < .001) and disulfide/native thiol (*P* < .001) ratios were markedly augmented in the A-CD group (Figure 4). These ratios were not significantly different in the R-CD group compared to controls (*P* > .05 for all).

Significant correlations between oxidative/nitrosative stress parameters in patients with A-CD at onset are demonstrated in Table 2. Although significant positive correlations were recorded between 3-NT versus GSH-Px, GSH, and MPO, negative correlations were documented between native thiol versus disulfide, and GSH-Px versus NO levels (Table 2). In R-CD patients, 3-NT levels were also positively correlated with GSH-Px, GSH, and MPO. Interestingly, smoking was negatively correlated with 3-NT, GSH-Px, and GSH levels (Table 3). Importantly, no significant correlations were found between clinical disease activity and any of the measured oxidative/nitrosative stress parameters.

## Discussion

This study highlights the critical role of oxidative/nitrosative stress and dynamic thiol/disulfide homeostasis in CD. Our data demonstrated that serum levels of the native thiol were significantly depressed during the active phases of CD in patients. Furthermore, we observed elevated serum NO levels and diminished 3-NT levels in CD patients compared to controls.

Growing evidence suggests that inflammation and exaggerated mucosal damage in the early phase of CD are linked with the infiltration of stimulated inflammatory cells.^1,5^ Stimulated neutrophils play a pivotal role in the recruitment of activated immune cells and generate excess MPO, ROS/RNS, and pro-inflammatory cytokines, leading to tissue damage.^1,5^ Elevated ROS levels associated with mucosal damage trigger oxidative stress. Notably, significant correlations exist between CD disease activity and increased mucosal ROS generation in the inflamed colon.^1^ Infiltration of activated neutrophils leads to an augmented risk of thrombosis and compromised resolution of intestinal inflammation during CD.^1,5^ These findings underscore the significant contribution of ROS to the pathophysiology of CD.

We have observed increased platelet counts in patients with active CD at onset. There is evidence showing that thrombocytosis occurs simultaneously with oxidative stress and inflammatory activity in CD.^10^ Regarding the inflammatory status, we observed meaningfully higher levels of CRP in active patients than in patients in the remission, which is consistent with the study published by Maor et al^11^

We have found elevated serum NO levels in both the active and remission phases of CD patients, and these levels were not significantly different between the A-CD and R-CD groups. Our results confirm findings from previous studies, such as the study by Oudkerk Pool et al^12^ who also observed significantly higher serum NO levels in CD patients in the active phase of the disease. Elevated NO production in the colonic mucosa of patients with active CD was reported.^13^ Overall, these results suggest that NO levels are consistently upregulated in CD.

NO regulates various physiological processes in the gastrointestinal tract. Beyond its well-established vasorelaxant effects, NO exhibits inhibition of neutrophils, suppression of adhesion and aggregation of platelets, dilation of nonvascular smooth muscle cells in response to stimulation of peptidergic nerves, augmentation epithelial permeability, and regulation of transepithelial ion secretion. Moreover, it has been suggested that NO may have protective effects during active inflammation.^14^ In colon biopsies obtained from patients with CD, augmented inducible nitric oxide synthase activity and increased NO production were identified.^14^ These findings suggest that at the site of inflammation, the colonic mucosal formation of NO is clearly elevated, and excessive amounts of NO could have cytotoxic effects on colonic cells through the formation of peroxynitrite in CD.

Earlier studies have repeatedly shown that IBD patients demonstrate a diminished total blood antioxidant status, as reflected by reduced total antioxidant capacity and low levels of antioxidant enzymes such as GSH-Px, superoxide dismutase (SOD), and catalase (CAT).^11^ However, findings regarding specific enzymatic antioxidants in plasma/erythrocytes remain somewhat contradictory. Some authors observed decreased^15^ or elevated^16^ levels of antioxidant enzymes in patients with active intestinal inflammation.

The role of SOD, which catalyzes the conversion of superoxide radicals into H_2_O_2_ and O_2_, remains unclear in CD. Decreased Cu/Zn-SOD activity and protein levels in peripheral blood cells of CD patients have been reported.^17^ In contrast, Kruidenier et al^18^ observed selective elevation of mitochondrial Mn-SOD levels in the mucosal tissue of CD patients. Additionally, Tavassolifar et al^19^ revealed significantly upregulated mRNA levels of Cu/Zn-SOD and CAT in CD patients compared to controls. However, owing to the fact that peroxynitrite is the coupling product of NO with superoxide, a decrease in superoxide due to increased SOD could lead to a decrease in peroxynitrite. This may explain decreased serum 3-NT levels in CD patients found in our study. In another report, Dijkstra et al^14^ documented that nitrotyrosine formation was observed on ROS-positive cells, but no differences in 3-NT immunoreactivity between non-inflamed or inflamed CD mucosa and normal controls were observed. These findings collectively suggest that superoxide, NO, and peroxynitrite contribute to CD pathogenesis.

H_2_O_2_ can be eliminated by 2 different enzymes: GSH-Px and CAT. GSH-Px is clearly related to the protection of cells from the damaging effects of ROS. It follows the action of SOD and catalyzes the decomposition of H_2_O_2_ into water and oxygen. Our results showed that there was a depressed GSH-Px in CD, and serum GSH-Px negatively correlated with NO levels, but our results do not support the previous studies reporting increased plasma GSH-Px activity in CD patients.^16,20^ Barros et al^21^ reported that erythrocyte GSH-Px activity was increased in the CD groups. Different studies have presented that GSH-Px is elevated in both plasma^18^ and the colon biopsies^16^ of active CD patients and is regarded as a defense mechanism against oxidative stress. Kruidenier et al^18^ observed a rise in GSH-Px activity in the intestinal CD mucosal tissue in comparison to the controls. Our results suggest that a compromised antioxidant state plays a pivotal role in both active and remission phases of CD.

We have determined diminished serum GSH levels in CD patients, which may support the findings in the affected and normal ileum of CD patients.^22^ Furthermore, our results agree with Szczeklik et al,^23^ who reported decreased serum GSH levels in both inactive and active CD groups. The inflamed ileum of patients with active CD is characterized by both a reduction of GSH and an augmentation of oxidized glutathione (GSSG, glutathione disulfide) in tissue samples obtained from the terminal ileum.^23,24^ Serum GSH levels were also found to be significantly decreased.^25^ The GSH-Px activity, as well as the concentration of its cofactor GSH, has been reported to drop in inflamed mucosa.^23,24^ Similarly, Ruan et al^26^ documented lower GSH levels in inflamed intestinal mucosa compared to healthy controls, with no significant difference between inflamed and non-inflamed mucosa in CD patients. Collectively, these findings suggest that an elevated consumption of GSH in response to nitrosative/oxidative stress may result in decreased cellular GSH levels.

GSH plays a fundamental role in cellular functions, changing the activities of various proteins through thiol-disulfide reactions. It is an important component of the overall antioxidant defense due to its radical scavenging properties. When thiol/disulfide homeostasis shifts towards disulfide production, cellular vital activities are inversely influenced, and this may lead to pathological changes. Available data regarding thiol/disulfide homeostasis in CD is scarce. We have demonstrated that serum native and total thiol levels were attenuated in active CD patients at onset but not in CD patients in the remission. In other words, there were no changes in serum free thiols in CD patients in the remission compared to controls. We also demonstrated augmented disulfide levels in CD. Similar to our results, Bourgonje et al^6^ indicated that free thiol levels in plasma were significantly declined in active CD patients compared to healthy controls. However, our data was not in agreement with Bourgonje et al^6^ who demonstrated that plasma free thiols were diminished in patients with CD in clinical remission compared to controls. Reduced plasma thiol levels were also demonstrated in other studies.^27^ Yuksel et al^28^ indicated that the diminished levels of thiol and elevated levels of disulfide were detected in the IBD group compared to the control group, which is in accordance with our study. We suggest that decreased levels of thiol in patients with active CD are due to increased oxidation.

MPO reacts with H_2_O_2_ and chloride ions to produce hypochlorous acid, a potent oxidizing substance. It has also been indicated that hypochlorous acid is much more toxic than common ROS and is able to interact easily with amino acids, sulfhydryls, pyridine nucleotides, and nitrogenous compounds.^29^ Swaminathan et al^29^ reported that fecal myeloperoxidase can be used as a biomarker of endoscopic disease activity in CD. Interestingly, our study reveals decreased serum MPO levels in CD patients.

Epidemiological evidence strongly implicates cigarette smoking as a major risk factor for CD, both increasing susceptibility and worsening disease progression.^30^ To investigate the potential impacts of smoking on oxidative/nitrosative stress, we conducted correlation analyses. Results revealed negative correlations between smoking and serum 3-NT, GSH-Px, and GSH levels, specifically in CD patients in the remission. This correlation was not observed in patients with active CD at onset.

This study has several limitations. First, we could not directly compare active and remission states within the same patient. Second, most of the patients in the remission phase were also taking medications, and this may affect the results. In the present study, several agents (5-aminosalicylic acid, azathioprine, 6-mercaptopurine, mesalazine, adalimumab, certolizumab, infliximab, and etanercept) were used to put Crohn’s patients into remission. Medications were administered according to individualized treatment needs and indications. Since all the patients received drug combinations, the potential influence of single medications on the measured parameters could not be evaluated. Thirdly, endoscopic activity assessments were not feasible for all patients. Finally, our sample size was relatively small. Future studies with larger cohorts will be necessary to provide a more comprehensive understanding of the biochemical characteristics of this disease.

In conclusion, the current study demonstrates increased oxidative stress, diminished antioxidative status, elevated NO levels, and depressed thiol levels in CD. These findings suggest that serum thiol/disulfide homeostasis could be used as a non-invasive, cost-effective biomarker of CD. The monitoring of thiol/disulfide homeostasis could help discriminate between the discrimination of active and remission phases of the disease. Our data reveal a shift in thiol-disulfide equilibrium towards the oxidative state, accompanied by a depletion of the key antioxidant GSH. This highlights the potential therapeutic value of thiol-containing drugs for CD management. Overall, incorporating antioxidant therapy into treatment regimens may provide a valuable strategy to improve disease outcomes in CD patients.

## Availability of Data and Materials:

The data that support the findings of this study are available on request from the corresponding author.

## Figures and Tables

**Table 1.** Demographic and Laboratory Data of Patients with Active CD at Onset (A-CD), CD Patients in the Remission (R-CD), and Healthy Controls

	Control Group (n = 38)	A-CD (n = 38)	R-CD (n = 38)	*P* Value
Age (years)	35.3 ± 8.3	33.9 ± 11.4	39.1 ± 13.4	.1143
Gender Male (n %) Female (n %)	21 (55.3) 17 (44.7)	25 (65.8) 13 (34.2)	24 (63.2) 14 (36.8)	.6181
Current smoking Yes (n, %) No (n, %)	14 (36.8) 24 (63.2)	22 (57.9) 16 (42.1)	20 (52.6) 18 (47.4)	.1612
White blood cells (x10^3^/μl)	8.1 ± 1.4	9.4 ± 3.3	8.7 ± 3.1	.0897
Platelet count (x10^3^/μl)	301.4 ± 48.1	346.5 ± 98.6	299.6 ± 94.4	<.05* <.05***
MCV (fl)	87.0 ± 3.8	84.3 ± 67	87.2 ± 7.3	.0832
Hemoglobin (g/dl)	13.9 ± 1.8	13.0 ± 2.3	13.8 ± 1.7	.0689
ESR (mm/h)	13.7 ± 6.8	22.8 ± 9.8	24.5 ± 12.9	<.001* <.001**
C-reactive protein (mg/l)	1.8 ± 1.5	7.8 ± 7.3	5.8 ± 5.7	<.001* <.01**
Glucose (mg/dl)	87.6 ± 11.2	90.5 ± 17.5	94.6 ± 12.4	.0916
Urea (mg/dl)	24.9 ± 7.0	25.9 ± 8.0	25.0 ± 8.4	.8263
Creatinine (mg/dl)	0.7 ± 0.2	0.7 ± 0.4	0.7 ± 0.2	.5967
AST (IU/l)	26.2 ± 8.3	22.5 ± 7.8	23.1 ± 6.4	.0820
ALT (IU/l)	22.4 ± 11.7	21.3 ± 11.9	23.2 ± 12.7	.7799
ALP (IU/l)	57.9 ± 18.8	87.2 ± 22.5	81.9 ± 20.0	<.001* <.001**
GGT (IU/l)	21.4 ± 12.7	28.1 ± 16.0	26.2 ± 15.0	.1282
Iron (μg/dl)	102.4 ± 27.9	63.9 ± 29.9	58.5 ± 32.2	<.001* <.001**
TIBC (μg/dl)	350.2 ± 58.6	333.5 ± 66.3	292.1 ± 59.8	<.001** <.01***
Ferritin (ng/ml)	49.1 ± 33.1	39.2 ± 23.4	40.5 ± 35.3	.3210
Vitamin B_12_ (pg/ml)	287.8 ± 99.9	263.5 ± 101.8	235.8 ± 82.7	.0611
Folic acid (ng/ml)	8.1 ± 2.8	6.0 ± 3.1	6.7 ± 3.3	.0738
Albumin (g/l)	43.1 ± 2.3	40.1 ± 5.6	38.8 ± 3.5	<.01* <.001**
Serum globulin (g/l)	29.9 ± 2.7	31.0 ± 5.5	30.4 ± 5.4	.5652

Data show mean ± SD values; MCV, mean corpuscular volume; ALT, alanine aminotransferase; AST, aspartate aminotransferase; ALP, alkaline phosphatase; ESR, erythrocyte sedimentation rate; GGT, gamma-glutamyl transferase; TIBC, total iron-binding capacity. * Control group versus A-CD, ** Control group versus R-CD, *** A-CD versus R-CD.>

**Table 2.** Significant Correlations Between Oxidative and Nitrosative Stress Parameters in Patients with Active CD at Onset (A-CD)

Parameters	Correlation Coefficient (r)	Coefficient of Determination (r^2^)	*P* Value
Total thiol ⟷ Disufide	0.8645	0.7474	<.0001
Native thiol ⟷ Disulfide	−0.3422	0.1171	.0381
GSH-Px ⟷ NO	−0.4763	0.2269	.0033
GSH-Px ⟷ 3-NT	0.5871	0.3446	.0002
GSH ⟷ 3-NT	0.6657	0.4431	<.0001
MPO ⟷ 3-NT	0.7965	0.6344	<.0001
GSH-Px ⟷ GSH	0.6940	0.4817	<.0001
GSH-Px ⟷ MPO	0.8031	0.6450	<.0001
GSH ⟷ MPO	0.4684	0.2194	.0035

NO, nitric oxide; 3-NT, 3-nitrotyrosine; GSH-Px, glutathione peroxidase; GSH, glutathione; MPO, myeloperoxidase

**Table 3.** Significant Correlations Between Oxidative and Nitrosative Stress Parameters in CD Patients in Remission (R-CD)

Parameters	Correlation Coefficient (r)	Coefficient of Determination (r^2^)	*P* value
Native thiol ⟷ Total thiol	0.7033	0.4946	<.0001
Total thiol ⟷ Disulfide	0.5009	0.2509	.0014
GSH-Px ⟷ 3-NT	0.8892	0.7906	<.0001
GSH ⟷ 3-NT	0.9396	0.8828	<.0001
MPO ⟷ 3-NT	0.8709	0.7585	<.0001
GSH-Px ⟷ GSH	0.9429	0.8891	<.0001
GSH-Px ⟷ MPO	0.8588	0.7375	<.0001
GSH ⟷ MPO	0.9482	0.8990	<.0001
Smoking ⟷ 3-NT	−0.2437	0.0594	.0339
Smoking ⟷ GSH-Px	−0.2296	0.0527	.0475
Smoking ⟷ GSH	−0.2383	0.0568	.0382

GSH, glutathione; GSH-Px, glutathione peroxidase; MPO, myeloperoxidase; NO, nitric oxide; 3-NT, 3-nitrotyrosine.

## References

[1] Alemany-Cosme E Sáez-González E Moret I , et al. Oxidative stress in the pathogenesis of Crohn’s disease and the interconnection with immunological response, microbiota, external environmental factors, and epigenetics. Antioxidants (Basel). 2021;10(1):64. (10.3390/antiox10010064) 33430227 PMC7825667

[2] Törüner M Ünal NG . Epigenetics of inflammatory bowel diseases. Turk J Gastroenterol. 2023;34(5):437 448. (10.5152/tjg.2023.22515) 37158530 PMC10334590

[3] Kayar Y Dertli R Konür Ş , et al. Mucocutaneous manifestations and associated factors in patients with Crohn’s disease. Turk J Gastroenterol. 2022;33(11):945 954. (10.5152/tjg.2022.21750) 36098365 PMC9797716

[4] Ng SC Shi HY Hamidi N , et al. Worldwide incidence and prevalence of inflammatory bowel disease in the 21st century: a systematic review of population-based studies. Lancet. 2017;390(10114):2769 2778. (10.1016/S0140-6736(17)32448-0) 29050646

[5] Guan G Lan S . Implications of antioxidant systems in inflammatory bowel disease. BioMed Res Int. 2018;2018:1290179. (10.1155/2018/1290179) 29854724 PMC5966678

[6] Bourgonje AR Gabriëls RY de Borst MH , et al. Serum free thiols are superior to fecal calprotectin in reflecting endoscopic disease activity in inflammatory bowel disease. Antioxidants (Basel). 2019;8(9):351. (10.3390/antiox8090351) 31480545 PMC6769968

[7] Lennard-Jones JE . Classification of inflammatory bowel disease. Scand J Gastroenterol Suppl. 1989;170:2 19. (10.3109/00365528909091339) 2617184

[8] Harvey RF Bradshaw JM . A simple index of Crohn’s-disease activity. Lancet. 1980;1(8167):514. (10.1016/s0140-6736(80)92767-1) 6102236

[9] Yücel EM Konduk BT Saracaloglu A , et al. Investigation of dynamic thiol/disulfide homeostasis and nitrosative stress in patients with Wilson disease. Turk J Gastroenterol. 2021;32(9):765 773. (10.5152/tjg.2021.20549) 34609306 PMC8975326

[10] Li L Xu P Zhang Z Zhou X Chen C Lu C . Platelets can reflect the severity of Crohn’s disease without the effect of anemia. Clinics (Sao Paulo). 2020;75:e1596. (10.6061/clinics/2020/e1596) 32667493 PMC7337217

[11] Maor I Rainis T Lanir A Lavy A . Oxidative stress, inflammation and neutrophil superoxide release in patients with Crohn’s disease: distinction between active and non-active disease. Dig Dis Sci. 2008;53(8):2208 2214. (10.1007/s10620-007-0141-6) 18253831

[12] Oudkerk Pool M Bouma G Visser JJ , et al. Serum nitrate levels in ulcerative colitis and Crohn’s disease. Scand J Gastroenterol. 1995;30(8):784 788. (10.3109/00365529509096328) 7481547

[13] Soufli I Hablal A Bessaad S , et al. Nitric oxide, neutrophil/lymphocyte, and platelet/lymphocyte ratios as promising inflammatory biomarkers in complicated Crohn’s disease: outcomes of corticosteroids and Anti-TNF-α therapies. Inflammation. 2023;46(3):1091 1105. (10.1007/s10753-023-01796-4) 36869975

[14] Dijkstra G Moshage H van Dullemen HM , et al. Expression of nitric oxide synthases and formation of nitrotyrosine and reactive oxygen species in inflammatory bowel disease. J Pathol. 1998;186(4):416 421. (10.1002/(SICI)1096-9896(199812)186:4<416::AID-PATH201>3.0.CO;2-U) 10209492

[15] Reimund JM Hirth C Koehl C Baumann R Duclos B . Antioxidant and immune status in active Crohn’s disease. A possible relationship. Clin Nutr. 2000;19(1):43 48. (10.1054/clnu.1999.0073) 10700533

[16] Tüzün A Erdil A Inal V , et al. Oxidative stress and antioxidant capacity in patients with inflammatory bowel disease. Clin Biochem. 2002;35(7):569 572. (10.1016/s0009-9120(02)00361-2) 12493587

[17] Verspaget HW Peña AS Weterman IT Lamers CB . Diminished neutrophil function in Crohn’s disease and ulcerative colitis identified by decreased oxidative metabolism and low superoxide dismutase content. Gut. 1988;29(2):223 228. (10.1136/gut.29.2.223">10.1136/gut.29.2.223) 2831119 PMC1433298

[18] Kruidenier L Kuiper I van Duijn W , et al. Differential mucosal expression of three superoxide dismutase isoforms in inflammatory bowel disease. J Pathol. 2003;201(1):7 16. (10.1002/path.1407) 12950012

[19] Tavassolifar MJ Changaei M Salehi Z , et al. Redox imbalance in Crohn’s disease patients is modulated by azathioprine. Redox Rep. 2021;26(1):80 84. (10.1080/13510002.2021.1915665) 33882797 PMC8079067

[20] Dincer Y Erzin Y Himmetoglu S Gunes KN Bal K Akcay T . Oxidative DNA damage and antioxidant activity in patients with inflammatory bowel disease. Dig Dis Sci. 2007;52(7):1636 1641. (10.1007/s10620-006-9386-8) 17393334

[21] Barros SÉL Dias TMDS Moura MSB , et al. Relationship between selenium status and biomarkers of oxidative stress in Crohn’s disease. Nutrition. 2020;74:110762. (10.1016/j.nut.2020.110762) 32244179

[22] Iantomasi T Marraccini P Favilli F Vincenzini MT Ferretti P Tonelli F . Glutathione metabolism in Crohn’s disease. Biochem Med Metab Biol. 1994;53(2):87 91. (10.1006/bmmb.1994.1062) 7710773

[23] Szczeklik K Krzyściak W Cibor D , et al. Evaluation of plasma concentrations of selected antioxidant parameters in patients with active Crohn’s disease. Folia Med Cracov. 2018;58(2):119 130. (10.24425/fmc.2018.124663) 30467439

[24] Sido B Hack V Hochlehnert A Lipps H Herfarth C Dröge W . Impairment of intestinal glutathione synthesis in patients with inflammatory bowel disease. Gut. 1998;42(4):485 492. (10.1136/gut.42.4.485) 9616308 PMC1727080

[25] Abbas ZK Zaidan TF . Oral findings, oxidative stress and antioxidant biomarker assessment in serum and saliva of Crohn’s patients. Int J Sci Res. 2017;6:1684 1687.

[26] Ruan EA Rao S Burdick JS , et al. Glutathione levels in chronic inflammatory disorders of the human colon. Nutr Res. 1997;17(3):463 473. (10.1016/S0271-5317(97)00007-9)

[27] Passos RA Costa PRF da Maia Lima CF , et al. Thiols as a marker of inflammatory bowel disease activity: a systematic review. BMC Gastroenterol. 2023;23(1):94. (10.1186/s12876-023-02711-9) 36977983 PMC10052829

[28] Yuksel M Ates I Kaplan M , et al. The dynamic thiol/disulphide homeostasis in inflammatory bowel disease and its relation with disease activity and pathogenesis. Int J Colorectal Dis. 2016;31(6):1229 1231. (10.1007/s00384-015-2439-8) 26561415

[29] Swaminathan A Borichevsky GM Edwards TS , et al. Faecal myeloperoxidase as a biomarker of endoscopic activity in inflammatory bowel disease. J Crohns Colitis. 2022;16(12):1862 1873. (10.1093/ecco-jcc/jjac098) 35803583 PMC9721461

[30] Chen Y Wang Y Shen J . Role of environmental factors in the pathogenesis of Crohn’s disease: a critical review. Int J Colorectal Dis. 2019;34(12):2023 2034. (10.1007/s00384-019-03441-9) 31732875

